# Dental antibiotic stewardship: study protocol for developing international consensus on a core outcome set

**DOI:** 10.1186/s13063-022-06038-w

**Published:** 2022-02-04

**Authors:** Wendy Thompson, Leanne Teoh, Céline Pulcini, David Williams, Carole Pitkeathley, Vanessa Carter, Susie Sanderson, Glauco Torres, Tanya Walsh

**Affiliations:** 1 Division of Dentistry, University of Manchester, Manchester, UK; 2 Melbourne Dental School, University of Melbourne, Carlton, Victoria Australia; 3 Université de Lorraine, APEMAC, Nancy, France; 4 Institute of Dentistry, Queen Mary University of London, London, UK; 5 Blythe, UK; 6 Torquay, UK; 7 British Dental Association, London, UK; 8 London, UK

**Keywords:** Antibiotic, Dental, Prescribing, Resistance, Stewardship, Core outcome set

## Abstract

**Background:**

Antimicrobial resistance is both a global public health and patient safety problem driven by overprescribing of antibiotic and other antimicrobial drugs. To conserve the effectiveness of antibiotics for future generations, antibiotic stewardship approaches to using them only where appropriate and necessary are advocated. Dentistry accounts for about 10% of antibiotic prescriptions across global healthcare, with 80% not in accordance with guidance in some countries. Core outcome sets enable the results of studies to be compared in order to maximise the value which can be derived from them. The aim of this study is to develop an international consensus on a core outcome set for dental antibiotic stewardship.

**Methods:**

Consensus on outcomes which are critical for inclusion in the core outcome set for dental antibiotic stewardship will be sought through two rounds of a Delphi survey (using the DelphiManager online system) followed by a final online consensus meeting. Thirty participants will be recruited to the Delphi Panel from across three stakeholder groups: ten dentists, ten academics and ten adults experienced with dental antibiotics as either a patient or parent/carer of a patient who has been prescribed them. Consensus will be achieved if more than 70% of the panel agree that an outcome is critical, with at least one from each stakeholder group in agreement. A long-list of candidate core outcomes has been developed from previously published studies with additions recommended by the steering group. The steering group will oversee development of the core outcome set and includes people from around the world with experience of dental antibiotics: clinicians, researchers and people with experience of being prescribed dental antibiotics and/or surviving an antibiotic resistant infection.

**Discussion:**

To date, few studies of dental antibiotic stewardship have been published. Internationally, dental antibiotic guidelines and patterns of use vary widely, so a core outcome set is particularly important to facilitate meaningful comparisons between studies. This core outcome set will encompass antibiotic prescribing for both therapeutic indications, such as for people with acute infections, and for prophylactic indications, such as the prevention of distant site infections (like infective endocarditis) following dental procedures.

**Supplementary Information:**

The online version contains supplementary material available at 10.1186/s13063-022-06038-w.

## Introduction

### Background and objectives

Considered one of the biggest threats to global health by the World Health Organization, antimicrobial resistance is expected to be responsible for ten million deaths each year by 2050 [1]. It is both a global public health and patient safety problem driven by overprescribing of antimicrobial drugs. To conserve the effectiveness of antimicrobials for future generations, the World Health Organization advocates using them only where appropriate and necessary [1]. Antimicrobial stewardship aims to ensure sustainable access to effective therapy for all who need them by minimising unnecessary and inappropriate prescribing [2]. Antibiotics (antibacterial drugs) are the antimicrobials most prescribed by dentists [2]. Dentists are responsible for an estimated 10% of antibiotics across healthcare worldwide, with most in primary dental care and outpatient settings, and up to 80% not in accordance with guidelines [3].

Whilst a plethora of trials of antibiotic stewardship interventions have been successfully conducted in primary and secondary care medical settings, few have so far taken place in dental care settings [4, 5]. Internationally, there is growing recognition of the essential role the dental profession can play in efforts to tackle antibiotic resistance through antibiotic stewardship [3]. As a result, FDI World Dental Federation has recently established an early career researcher network: Global Antimicrobial Resistance Dentistry early career researcher network, to nurture a research base for dental antimicrobial prescribing, stewardship and resistance [6].

Dental antibiotic guidelines and patterns of use are known to vary widely, and efforts to compare figures for dental antibiotic use and appropriate prescribing between countries have been hampered by differences in the ways data are collected and dental services are delivered [7]. Guideline differences are particularly important, meaning that in some countries prophylactic use of dental antibiotics to prevent distance site infection is most common (such as in the USA for the prophylaxis of infective endocarditis), whereas in other countries, therapeutic use for the treatment of infections predominates (for example in the UK) [3, 7]. As a result of these developments, it is anticipated that there will be an increase in the number of dental antibiotic stewardship studies. Establishing an international consensus on outcomes before the anticipated growth occurs will ensure the benefit derivable from them is maximised.

Core outcome sets (COS) are an agreed standardised set of outcomes that should be measured and reported by all studies in a specific area [8]. COS allow meaningful collation of results across multiple institutions internationally by reducing both heterogeneity between studies, care settings and outcome reporting bias. To meet the aims of specific studies or care contexts, core sets are supplemented with additional measures when used in practice.

International consensus on a core outcome set for dental antibiotic stewardship is particularly important to facilitate meaningful comparisons of dental antibiotic prescribing between countries and to assist global efforts to tackle antibiotic resistance.

The objective of this study is to develop an international consensus on a core outcome set for dental antibiotic stewardship (COS-DABS). Further studies will be required to identify outcome measurement instruments.

### Scope

COS-DABS will cover both therapeutic and prophylactic prescribing of antibiotics by dentists in primary dental care and community dental settings. It will be designed for use in clinical trials to evaluate antibiotic stewardship interventions.

## Methods

The study will be carried out in accordance with the COMET guidance [9]. This protocol is presented using the Core Outcome Set-STAndardised Protocol (COS-STAP) Items Statement (see Supplementary Material Table 1) [8].

### Stakeholders

This study will involve online participation of people with experience of dental antibiotics from around the world. There is no restriction on which countries will be involved nor the setting in which the participants have experience of dental antibiotics.

Inclusion criteria for participating in this study will be people over 18 years who are able to give informed consent and who have experience of dental antibiotics as members of one of the following stakeholder groups:

- Clinicians with experience of dental antibiotic prescribing;
- Academics with an interest in dental antibiotic prescribing; or
- People over 18 years of age who have taken antibiotics for a dental (therapeutic or prophylactic) reason or the parents/carers of people who have taken dental antibiotics.

Anyone under the age of 18 years, those who are not able to give informed consent and those with no experience of dental antibiotics will be excluded.

A total of 30 stakeholders will be recruited to participate in the study. This was a pragmatic choice taking account of the relatively small size of the pool of clinical and academic experts relating to dental antimicrobial stewardship [9]. There will be ten clinicians, ten academics and ten patients/parents/carers (as outlined above).

Convenience sampling of clinicians will be through their membership of relevant societies or organisations (such as national dental associations) or through social media (Twitter). Convenience sampling of academic stakeholders will be through the FDI World Dental Federation early career researcher network [6], social media (Twitter) and authors with relevant publications in the field of dental antibiotic stewardship [10]. Adult patients and parent/carer participants will be recruited by convenience sampling through patient representative organisations, social media (Twitter) and via members of the steering group.

### Steering group

A steering group has overseen development of the study protocol, including the long-list of candidate outcomes which will form the basis of the Delphi survey. It will oversee implementation of the protocol towards the development of COS-DABS.

The steering group consists of clinical academic dentists (WT and LT), consultants in global oral health (DW) and infectious diseases (CPu), an academic with experience of COS development (TW), a national dental association representative (SS) and three experts by experience of taking antibiotics prescribed by a dental professional (VC, CPi and GT). Its members are Australian, Brazilian, British, French and South African. They came together through involvement with the FDI World Dental Federation and British Society for Antimicrobial Chemotherapy’s collaborative project to develop an online course about the role of dental teams tackling antibiotic resistance in 2020 [3]. All members of the steering group will be co-authors of the COS-DABS final report.

The three experts by experience of dental antibiotics and/or complications of antibiotics were recruited to the steering group via a European patient representative body (EUPATI), a charity focused on antimicrobial chemotherapy (British Society for Antimicrobial Chemotherapy), and from the coproduction team of a systematic review contributed to the long-list of candidate outcomes for this study [11]. The patient experience of antibiotic prescribing and resistance has informed and will continue to inform all stages of this study.

### Information sources

A long-list of candidate outcomes for inclusion in the consensus exercise has been produced (see Table 1) based on findings of previously published studies [10–12] and structured according to a review evaluating antimicrobial stewardship interventions across healthcare [13]. Additional items for inclusion in the long-list were identified by the study steering group.

**Table 1:** Nine-point Likert scale for participants to rate the candidate outcomes

Outcome	Not important			Important but not critical			Critical			
	1	2	3	4	5	6	7	8	9	Unable to rate
**Antibiotic use**										
Amount (e.g. number) of antibiotics prescribed										
Rate of antibiotic prescribing										
Appropriateness of antibiotic prescribing										
**Complications or harm**										
Complications or harm resulting from antibiotic treatment										
Complications or harm resulting from disease progression										
Complications of harm resulting from surgical site (wound) infection										
Complications or harm resulting from distance site infection (elsewhere in the body)										
Need for escalation of care										
Serious adverse outcomes										
**Patient-reported measures**										
Satisfaction with the result (outcome) of the care provided										
Satisfaction with the dental treatment provided										
Need to taking time off usual responsibilities										
Mental health impact										
Ability to carry on with daily life as normal										
**Cost of the intervention**										
Cost to the healthcare system										
Cost to dental prescribers										
Cost to patients										
**Time to clinical response (for studies of therapeutic dental antibiotic prescribing)**										
Time taken until treatment										
Time until symptom resolution (after treatment)										
Severity of symptoms whilst waiting for resolution (after treatment)										
Number of unplanned return dental visits (after treatment)										

The first of the previously published studies was a hybrid umbrella/systematic review of antibiotic stewardship measures used across primary healthcare settings [10], which found few studies of antibiotic stewardship interventions in dentistry. Furthermore, the measures used in those dental studies focused exclusively on antibiotic use. By contrast, across primary medical care, more studies were identified and these employed a wider range of outcome measures, including complications (e.g. allergy to antibiotics) and patient satisfaction.

The second study was a systematic review of outcomes of care for adults with acute dental pain and infection [11]. This research was coproduced with experts by experience of dental antibiotics. As reported also in the antibiotic stewardship hybrid review [10], the outcomes for acute dental pain/infection which were reported to have been measured in dental settings focused more narrowly on clinical outcomes, compared to studies in non-dental settings (such as pharmacies and hospital emergency departments).

The third study included was an international consensus on a standardised core set of oral health outcome measures for adults [12]. Whilst this outcome set had a particular emphasis on caries and periodontal disease for use in clinical practice and population health, the study steering group wished to consider whether any of its measures were also applicable for COS-DABS, to ensure continuity, where possible, of outcome sets across dentistry.

Resolution of conflicts about which outcomes to include on the long-list of candidate outcomes was resolved by discussion among the steering group.

### Consensus process

Consensus on a core outcome set will be sought through two rounds of an online Delphi survey using the DelphiManager system [9, 14]. A nine-point Likert scale will be used by participants to rate the importance of dental antibiotic stewardship outcomes from the long-list of candidate outcomes (Table 1). At the end of the first round, participants will be invited to propose additional outcomes for inclusion in the long-list of candidate outcomes. Potential additions to the long-list, suggested by at least two participants in round 1, will be included for rating in round 2.

Rating each outcome will involve participants picking a number between 1 and 9 to signify importance. On this scale, a score of 1-3 indicates 'limited importance', 4-6 indicates 'important' and 7-9 signifies 'critically important' outcomes. There will also be an option for participants to select: ‘Unable to rate’ for each outcome.

All participants who participate in both rounds of the survey will be invited by email to take part in a final online consensus meeting. To be quorate, the meeting will need to comprise at least two people from each stakeholder group (clinicians, academics and patients/parents/carers).

### Consensus definition

Outcomes will be included in the final COS if they are voted for by at least 70% of participants, including at least one from each stakeholder group (clinician, academic or patient/parent/carer) [15]. Any outcome rated as ‘critical’ by fewer than 50% and ‘unimportant’ by more than 70% will be excluded from the COS [16]. All other combinations indicate that no consensus had been achieved for the outcome. Where necessary to address specific issues, it will be possible for the stakeholder groups at the final consensus meeting to have separate discussions in ‘break out groups’ facilitated by a member of the study steering group. If more than 5 of the outcomes are rated as ‘critical’ for inclusion in the core set, the steering group may opt to apply a higher threshold of 75% and 25%, respectively.

## Analysis

### Outcomes scoring/feedback

In round 1, panel participants will be asked to score each long-listed outcome on a scale 1–9: with 1–3 identified as ‘not important’, 4–6 as ‘important but not critical’ and 7–9 labelled ‘critical’ [17]. Responses from round 1 will be summarised by stakeholder group, to show the distribution of scores recorded and median score for each item.

In round 2, panel participants will be asked to score all outcomes from round 1 plus additional outcomes suggested by at least two participants in round 1. Participants will be shown their previous individual score and asked to reconsider it in the light of descriptive statistics provided, relating to how other participants scored each candidate outcome. The descriptive statistics will comprise the median and the number of participants scoring the outcomes as critically important/important/limited importance votes for each stakeholder group (clinician, academic or patient/parent/carer).

To promote retention, two reminders will be sent to non-responding participants at the end of weeks 2 and 3 in each round of the Delphi survey.

### Missing data

Attrition (non-responders) and partial responses are two sources of missing data in a COS development exercise. To take account of attrition bias, distribution of the scores for those who participated only in round 1 will be compared with the equivalent round 1 data for those who went on to complete round 2. Changes in participant scores between rounds will be summarised along with the reasons given. Outcomes with missing data will be considered at the final consensus meeting to ensure inadvertent exclusion is avoided.

### Finalising the core outcome set

A meeting to review the final survey results and finalise COS-DABS by consensus will take place online via Zoom and in English language. All stakeholder groups of participants will be invited to the same meeting. No audio- or video-recording of the online meeting will be made. Presentation of the findings of the Round 2 Delphi will include the final list of outcomes and how they were voted for by each stakeholder group. A timed discussion will be moderated by an experienced facilitator to decide how to deal with any outcomes associated with missing data, where scores between stakeholder groups differed and for items scored only in round 2 (following identification in round 1 by more than one participant). Following discussion, there will be a vote on each outcome. Outcomes will be included in COS-DABS if they are voted for by at least 70% of participants, including at least one from each stakeholder group.

## Ethics and dissemination

### Ethics approval/informed consent

Ethical approval for the study was gained from the University of Manchester Research Ethics Committee proportional review process (ref UREC 2021-11905-20268 dated 02 August 2021 and amendment approved 06/09/2021). This study has also been registered with the COMET Initiative: https://comet-initiative.org/Studies/Details/1860.

The online survey in DelphiManager includes a consent checkbox for participants to confirm: ‘I agree to participate in, and receive email notifications regarding this study.’ All participants must check this box before they can gain access to the survey. Informed consent will be obtained from all participants via this online process at the start of round 1. For the final consensus meeting, logging into the online meeting will indicate consent to participate. This will be reiterated in the email invitation to the final consensus meeting and also verbally at the start of the meeting.

### Dissemination

The Core Outcome Set-STAndards for Reporting Equator Network guidelines will be used for the reporting of the COS [18]. All members of the steering group will co-author the final paper, which will be submitted for peer review and publication in an open access journal. To disseminate the findings as widely as possible, the results will be presented at international meetings in the dental and antimicrobial stewardship academic and clinical domains, including the International Association for Dental Research and World Dental Congress. In addition, there will be a final report to the funder.

## Trial status

Protocol v1 dated 08 September 2021. Recruitment is expected to begin in October 2021 and to be complete by December 2021.

## Supplementary Information

**Additional file 1: Supplementary Material Table 1.** COS-STAP items cross-referenced to manuscript subtitles.

## Data Availability

All data generated during the development of COS-DABS will be published in a peer-reviewed journal.
