# Supplementary material for: Dental antibiotic stewardship: study protocol for developing international consensus on a core outcome set
Source: Trials. 2022 Feb 4;23:116. doi: 10.1186/s13063-022-06038-w (PMC8817512; doi:10.1186/s13063-022-06038-w)
Supplement: Supplementary file 1 — Additional file 1: Supplementary Material Table 1. COS-STAP items cross-referenced to manuscript subtitles. [file 13063_2022_6038_MOESM1_ESM.docx]

Supplementary Material Table 1

**COS-STAP items cross-referenced to manuscript subtitles**

| **COS-STAP Item** | | **Section in manuscript** | **Page number in manuscript** |
| --- | --- | --- | --- |
| TITLE/ABSTRACT | | |  |
| Title | Title | | 1&2 |
| Abstract | Abstract | | 2-3 |
| INTRODUCTION | | |  |
| Background and objectives | Background and objectives | | 4-5 |
| Scope | Scope | | 5 |
| METHODS | | |  |
| Stakeholders | Stakeholders | | 6 |
| Information sources | Information sources | | 7-9 |
| Consensus process | Consensus process | | 9 |
| Consensus definition | Consensus definition | | 10 |
| ANALYSIS | | |  |
| Outcome scoring/feedback | Outcome scoring/feedback | | 10 |
| Missing data | Missing data | | 11 |
| ETHICS/DISSEMINATION | | |  |
| Ethics approval/informed consent | Ethics approval/informed consent | | 11-12 |
| Dissemination | Dissemination | | 12 |
| ADMINISTRATIVE INFORMATION | | |  |
| Funders | Funding | | 13 |
| Conflicts of interest | Competing interests | | 13 |
